# Bis(4-amino­pyridinium) μ_6_-oxido-dodeca-μ_2_-oxido-hexaoxido[rhenium(VII)tetratungsten(VI)vanadium(V)]ate hepta­hydrate

**DOI:** 10.1107/S1600536813030687

**Published:** 2013-11-16

**Authors:** Ahlem Maaloui, Samah Akriche Toumi, Mohamed Rzaigui

**Affiliations:** a Laboratoire de Chimie des Matériaux, Faculté des Sciences de Bizerte, 7021 Zarzouna Bizerte, Tunisia

## Abstract

In the title organic–inorganic hybrid compound, (C_5_H_7_N_2_)_2_[ReVW_4_O_19_]·7H_2_O, the Lindqvist-type polyoxido anion has crystallographically imposed *mm*2 symmetry and is built up by six *M*O_6_ (*M* = W, V, Re) edge-sharing distorted octa­hedra. The Re and V atoms share the same crystallographic site in a 0.5:0.5 ratio. The 4-amino­pyridinium cations lie on a mirror plane. Three of the four independent water O atoms in the asymmetric unit are located on a mirror plane whereas the remaining O atom has *mm*2 site symmetry. In the crystal, the cations, anions and water mol­ecules are linked into a three-dimensional network through O—H⋯O and N—H⋯O hydrogen-bonding inter­actions.

## Related literature

For applications of polyoxidometalates, see: Pope & Müller (1991 ▶, 1994 ▶). For bond-valence calculations, see: Brown & Altermatt (1985 ▶). For related structures, see: Lindqvist (1953 ▶); Bannani *et al.* (2007 ▶); Besecker *et al.* (1982 ▶); Wang *et al.* (2011 ▶); Wang *et al.* (2006 ▶); Meng *et al.* (2006 ▶). For NMR investigations of related compounds, see: Chen *et al.* (2004 ▶); Domaille (1984 ▶); Fedotov & Maksimovskaya (2006 ▶); Leparulo-Loftus & Pope (1987 ▶).
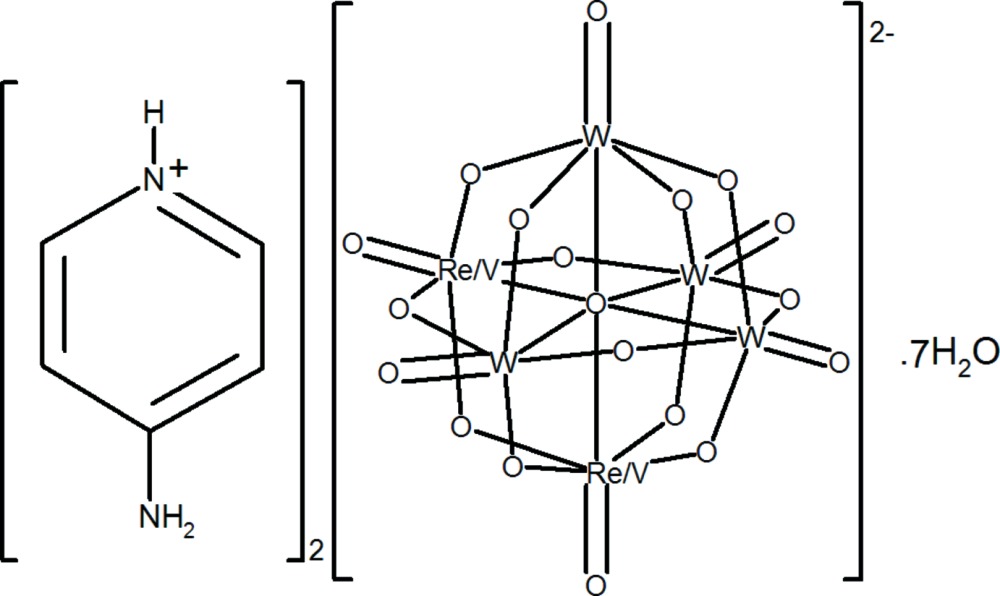


## Experimental

### 

#### Crystal data

(C_5_H_7_N_2_)_2_[ReVW_4_O_19_]·7H_2_O
*M* *_r_* = 1592.90
Orthorhombic, 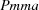

*a* = 17.837 (2) Å
*b* = 9.163 (4) Å
*c* = 10.552 (3) Å
*V* = 1724.5 (9) Å^3^
*Z* = 2
Ag *K*α radiation
λ = 0.56087 Å
μ = 9.22 mm^−1^
*T* = 298 K
0.43 × 0.27 × 0.15 mm

#### Data collection

Enraf–Nonius CAD-4 diffractometer
Absorption correction: multi-scan (Blessing, 1995 ▶) *T*_min_ = 0.100, *T*_max_ = 0.306
6539 measured reflections
4385 independent reflections
2083 reflections with *I* > 2σ(*I*)
*R*_int_ = 0.052
2 standard reflections every 120 min intensity decay: none

#### Refinement

*R*[*F* ^2^ > 2σ(*F* ^2^)] = 0.069
*wR*(*F* ^2^) = 0.192
*S* = 1.03
4385 reflections
138 parameters
17 restraints
H-atom parameters constrained
Δρ_max_ = 2.94 e Å^−3^
Δρ_min_ = −2.83 e Å^−3^

### 

Data collection: *CAD-4 EXPRESS* (Enraf–Nonius, 1994 ▶); cell refinement: *CAD-4 EXPRESS*; data reduction: *XCAD4* (Harms & Wocadlo, 1996 ▶); program(s) used to solve structure: *SHELXS97* (Sheldrick, 2008 ▶); program(s) used to refine structure: *SHELXL97* (Sheldrick, 2008 ▶); molecular graphics: *ORTEP-3 for Windows* (Farrugia, 2012 ▶) and *DIAMOND* (Brandenburg & Putz, 2005 ▶); software used to prepare material for publication: *WinGX* (Farrugia, 2012 ▶).

## Supplementary Material

Crystal structure: contains datablock(s) I. DOI: 10.1107/S1600536813030687/rz5088sup1.cif

Structure factors: contains datablock(s) I. DOI: 10.1107/S1600536813030687/rz5088Isup2.hkl

Additional supplementary materials:  crystallographic information; 3D view; checkCIF report

## Figures and Tables

**Table 1:** Hydrogen-bond geometry (Å, °)

*D*—H⋯*A*	*D*—H	H⋯*A*	*D*⋯*A*	*D*—H⋯*A*
N1—H1*A*⋯O1*W*	0.86	2.40	3.078 (14)	136
N1—H1*A*⋯O1*E*	0.86	2.58	3.184 (14)	129
N2—H2*A*⋯O3^i^	0.86	2.39	3.181 (12)	152
O1*W*—H1*W*1⋯O1*E*	0.81	2.50	3.188 (18)	144
O2*W*—H1*W*2⋯O3*E* ^ii^	0.85	2.10	2.836 (18)	144
O4*W*—H1*W*4⋯O2*W*	0.85	1.87	2.40 (7)	119
O4*W*—H2*W*4⋯O4*W* ^iii^	0.85	2.31	2.65 (15)	106

Symmetry codes: (i) 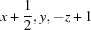; (ii) 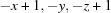; (iii) 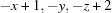.

**Fig. 1.**
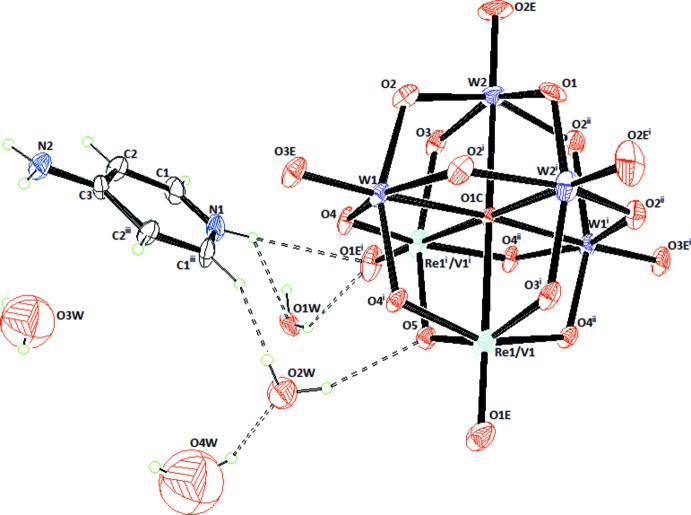
A view of the title compound with displacement ellipsoids drawn at the 30% probability level. H atoms are represented as small spheres of arbitrary radii. Hydrogen bonds are shown as dashed lines. Symmetry codes: (i) 1/2 + x, - y, 1 - z; (ii) 1/2 + x, y, 1 - z; (iii) x, 1 - y, z.

**Fig. 2.**
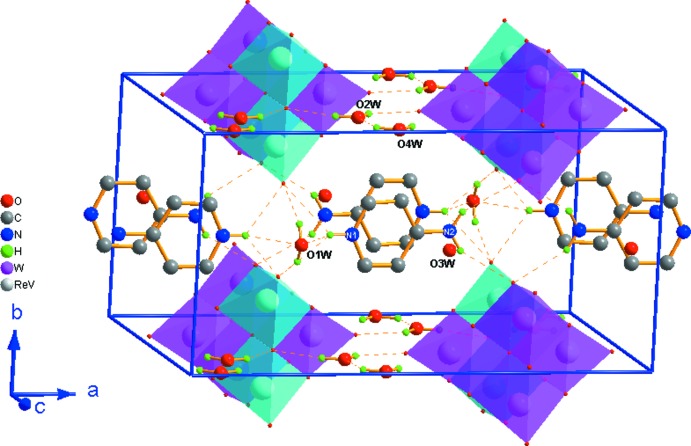
Packing diagram of the title compound. Hydrogen bonds are shown as dashed lines. Hydrogen atoms not involved in hydrogen bonding are omitted.

## supplementary crystallographic information

### 1. Comment

Polyoxidometalate are of great interest in different fields such as medecine, biology, catalysis, material sciences, chemical analysis (Pope & Müller, 1991; Pope & Müller, 1994). Among this large class of compounds, Lindqvist anions [*M*_6_O_19_]^n-^ have been intensively studied since the early characterization of Na_7_HNb_6_O_19_.15H_2_O (Lindqvist, 1953). However, studies of substituted hexatungstate [*M*_n_W_6-n_O_19_]^(2+n)-^ are relatively scarce. Up to now, few compounds are cited in the literature, such as [(n-C_4_H_9_)_4_N]_3_MW_5_O_19_ (*M* = Nb, V) (Bannani *et al.*, 2007), {[(C_7_H_8_)Rh]_5_(Nb_2_W_4_O_19_)_2_}[(n-C_4_H_9_)N]_3_ (Besecker *et al.*, 1982), [Cu(phen)(H_2_O)_3_]_2_[V_2_W_4_O_19_] and [Cu(bpy)(H_2_O)]_2_[V_2_W_4_O_19_]·4H_2_O (Wang *et al.*, 2011) and [Ni(bpy)_3_]_2_[W_4_V_2_O19] (Wang *et al.*, 2006), whereas substituted Lindqvist-type polyoxotungstates based on [*M*_n_M'_p_W_6-n-p_O_19_]^(2+n+p)-^ anions have not been reported hitherto. On the best of our knowledge the reported title salt, (C_5_H_7_N_2_)_2_·[ReVW_4_O_19_]·7H_2_O (I), is the first example of this kind of substituted Lindqvist structure.

The asymmetric unit of (I) contains 1.75 water molecules, one half of a 4-aminopyridinium cation and one fourth of a [ReVW_4_O_19_]^2-^ polyanion, in which Re and V metals share the same site in a 0.5:0.5 ratio. All constituents are completed by imposed crystallographic symmetries (Fig. 1). Three out of the four independent water O atoms in the asymmetric unit have mirror symmetry, while a fouth O atom has *mm*2 site symmetry. It is to be mentioned that the O3W and O4W oxygen atoms associated to water molecules have relatively high thermal desorder and could not be anisotropically refined. In the crystal packing, cations and solvent water molecules assemble the discrete [ReVW_4_O_19_]^2-^ anion complexes through OW—H···O and N—H···O hydrogen bonding interactions (Table 1) to form a supramolecular three-dimensional network as shown in Figure 2. The structure of the [ReVW_4_O_19_]^2-^ polyanion is basically the same as that of Lindqvist-type anion (Lindqvist, 1953). It is built upby six MO_6_ (*M* = W, V, Re) edge-sharing distorted octahedra and exhibits the characteristic M—O bond-length range, with the shortest bonds being the M—O terminal bonds and the longest being those involving the central O atom. The geometry features of the polyanion are in agreement with those of the Lindqvist-type polyoxidotungstate reported by Meng *et al.* (2006). The valence bond calculation (Brown & Altermatt, 1985) gives effective bond valences of 4.9847 for the V cation, 7.1447 for the Re cation and 6.0697 and 6.1369 for the two independent W cations. These values are consistent with the oxidation states V(V), Re(VII) and W(VI). In an attempt to shed more light on the structure of the hexametalate anion, we contemplated ^51^V and ^183^W NMR studies on the title complex. In fact, regarding the [*M*_6_O_19_]^n-^ complex structure, the vanadium and rhenium heteroatoms may occupy *cis* or *trans* positions in the octahedral structure. The ^183^W NMR study shows two signals with relative intensity of *ca* 2:2, which can be assigned to the two equatorial (Weq) and the two axial (Wax) tungsten atoms respectively. On the other hand, the ^51^V NMR spectrum presents one signal at 508.7 ppm. These results are consistent with the C_2v_ symmetry of the disubstitued hexametalate structure with preferential *cis* configuration as reported in literature (Chen *et al.*, 2004; Domaille, 1984; Fedotov & Maksimovskaya, 2006; Leparulo-Loftus & Pope, 1987). All these observations corroborate the structural results in suggesting that both rhenium and vanadium atoms (with 0.5/0.5 occupation) occupy preferentially the same site thus leading to a *cis*-[*X*_2_W_4_O_19_]^2-^ (*X* = Re_0.5_V_0.5_) anion configuration.

### 2. Experimental

The title compound was prepared by the reaction of vanadium(V) oxide (0.18 g, 1 mmol), rhenium(VII) oxide (0.48 g,1 mmol), Na_2_WO_4_.2H_2_O (2 g, 6 mmol) and 4-aminopyridine (0.19 g, 2 mmol) dissolved in 50 ml of distilled water and then stirred for 1 h. Yellowish single crystals were obtained after two weeks by slow evaporation at room temperature (yield: 59% based on W). Anal. Calc. for C_10_H_28_N_4_O_26_ReVW_4_: H 1.76, C 7.53, N 3.52, Re 11.69, V 3.20, W 46.16%; Found: H 1.81, C 7.57, N 3.50, Re 11.71, V 3.23, W 46.14%. ^183^W NMR δ (p.p.m.): 86.5, 65.4; ^51^V NMR δ (p.p.m.): 508.7.

### 3. Refinement

An initial attempt to refine the crystal structure with the Re atom disordered over three independent sites resulted in rather high atomic displacement parameters for the heaviest atoms (Re1, W1 and W2) as a possible consequence of thelarge number of restraints required by this model. The refinement of a model implying the Re atom sharing only the site occupied by the vanadium atom with an occupancy factor of 0.5 rapidly converged to a plausible result with low residuals. These observations are also supported by NMR study as detailed in Comment section. In spite the crystal selected for the X-ray analysis appeared to be of good quality, its diffraction ability was very poor. This may account for the rather high residual peaks, high R values and unresolved disorder affecting part of the water molecules. In fact, anisotropical refinement of the O atoms associated to water molecules resulted in unreasonable *U*_ij_ values for atoms O3W and O4W, which were therefore isotropically refined. The water H atoms could not be located and were placed geometrically sensible positions using restraints [O—H = 0.85 (1) Å, H···H = 1.44 (2) Å and *U*_iso_(H) = 1.5*U*_eq_(O). H atoms attached to C and N atoms were fixed geometrically and treated as riding, with C—H = 0.93 Å and N—H = 0.86 Å with *U*_iso_(H) = 1.2*U*_eq_(C, N). In the final difference Fourier map, the highest residual electron density peak and the deepest hole are located 0.78 and 0.64 Å respectively from W2.

### Crystal data

**Table:**

(C_5_H_7_N_2_)_2_[ReVW_4_O_19_]·7H_2_O	*F*(000) = 1436
*M**_r_* = 1592.90	*D*_x_ = 3.068 Mg m^−^^3^
Orthorhombic, *P**m**m**a*	Ag *K*α radiation, λ = 0.56087 Å
Hall symbol: -P 2a 2a	Cell parameters from 25 reflections
*a* = 17.837 (2) Å	θ = 9–11°
*b* = 9.163 (4) Å	µ = 9.22 mm^−^^1^
*c* = 10.552 (3) Å	*T* = 298 K
*V* = 1724.5 (9) Å^3^	Prism, yellow
*Z* = 2	0.43 × 0.27 × 0.15 mm

### Data collection

**Table:**

Enraf–Nonius CAD-4 diffractometer	2083 reflections with *I* > 2σ(*I*)
Radiation source: fine-focus sealed tube	*R*_int_ = 0.052
Graphite monochromator	θ_max_ = 28.0°, θ_min_ = 2.3°
non–profiled ω scans	*h* = −29→2
Absorption correction: multi-scan (Blessing, 1995)	*k* = −2→15
*T*_min_ = 0.100, *T*_max_ = 0.306	*l* = −17→2
6539 measured reflections	2 standard reflections every 120 min
4385 independent reflections	intensity decay: none

### Refinement

**Table:**

Refinement on *F*^2^	Primary atom site location: structure-invariant direct methods
Least-squares matrix: full	Secondary atom site location: difference Fourier map
*R*[*F*^2^ > 2σ(*F*^2^)] = 0.069	Hydrogen site location: inferred from neighbouring sites
*wR*(*F*^2^) = 0.192	H-atom parameters constrained
*S* = 1.03	*w* = 1/[σ^2^(*F*_o_^2^) + (0.0813*P*)^2^ + 4.9537*P*] where *P* = (*F*_o_^2^ + 2*F*_c_^2^)/3
4385 reflections	(Δ/σ)_max_ = 0.003
138 parameters	Δρ_max_ = 2.94 e Å^−^^3^
17 restraints	Δρ_min_ = −2.83 e Å^−^^3^

### Special details

**Table:**

Geometry. All e.s.d.'s (except the e.s.d. in the dihedral angle between two l.s. planes) are estimated using the full covariance matrix. The cell e.s.d.'s are taken into account individually in the estimation of e.s.d.'s in distances, angles and torsion angles; correlations between e.s.d.'s in cell parameters are only used when they are defined by crystal symmetry. An approximate (isotropic) treatment of cell e.s.d.'s is used for estimating e.s.d.'s involving l.s. planes.
Refinement. Refinement of *F*^2^ against ALL reflections. The weighted *R*-factor *wR* and goodness of fit *S* are based on *F*^2^, conventional *R*-factors *R* are based on *F*, with *F* set to zero for negative *F*^2^. The threshold expression of *F*^2^ > σ(*F*^2^) is used only for calculating *R*-factors(gt) *etc*. and is not relevant to the choice of reflections for refinement. *R*-factors based on *F*^2^ are statistically about twice as large as those based on *F*, and *R*- factors based on ALL data will be even larger.

### Fractional atomic coordinates and isotropic or equivalent isotropic displacement parameters (Å^2^)

**Table:**

	*x*	*y*	*z*	*U*_iso_*/*U*_eq_	Occ. (<1)
W1	0.37770 (3)	0.0000	0.34550 (7)	0.03479 (18)	
W2	0.2500	0.17748 (8)	0.18441 (8)	0.0497 (2)	
Re1	0.2500	0.18032 (13)	0.49071 (13)	0.0505 (3)	0.50
V1	0.2500	0.18032 (13)	0.49071 (13)	0.0505 (3)	0.50
O1C	0.2500	0.0000	0.3367 (12)	0.023 (2)	
O1E	0.2500	0.3071 (16)	0.6049 (16)	0.068 (4)	
O2E	0.2500	0.3115 (18)	0.0720 (17)	0.081 (5)	
O3E	0.4740 (6)	0.0000	0.3427 (13)	0.050 (3)	
O1	0.2500	0.0000	0.0922 (13)	0.048 (4)	
O2	0.3545 (4)	0.1420 (9)	0.2154 (9)	0.0466 (19)	
O3	0.2500	0.2888 (11)	0.3345 (12)	0.042 (3)	
O4	0.3529 (3)	0.1446 (8)	0.4650 (8)	0.0375 (16)	
O5	0.2500	0.0000	0.5902 (15)	0.039 (3)	
N1	0.3903 (7)	0.5000	0.6867 (14)	0.057 (4)	
H1A	0.3422	0.5000	0.6901	0.068*	
N2	0.6091 (7)	0.5000	0.6435 (17)	0.057 (4)	
H2A	0.6343	0.4198	0.649	0.069*	
C1	0.4219 (6)	0.6284 (14)	0.6845 (14)	0.056 (3)	
H1	0.3939	0.7134	0.6938	0.067*	
C2	0.4963 (6)	0.6327 (12)	0.6681 (13)	0.043 (3)	
H2	0.5214	0.7216	0.6642	0.052*	
C3	0.5363 (7)	0.5000	0.6570 (16)	0.038 (3)	
O1W	0.2500	0.5000	0.8564 (14)	0.063 (6)	
H1W1	0.2500	0.4226	0.8198	0.095*	
O2W	0.3772 (8)	0.0000	0.7518 (15)	0.079 (5)	
H1W2	0.410	0.0000	0.694	0.119*	
H2W2	0.332	0.0000	0.727	0.119*	
O3W	0.553 (3)	0.5000	0.960 (5)	0.27 (3)*	
H1W3	0.5644	0.5876	0.9741	0.402*	
O4W	0.429 (4)	0.0000	0.962 (6)	0.34 (3)*	
H1W4	0.386	0.0000	0.928	0.510*	
H2W4	0.466	0.0000	0.910	0.510*	

### Atomic displacement parameters (Å^2^)

**Table:**

	*U*^11^	*U*^22^	*U*^33^	*U*^12^	*U*^13^	*U*^23^
W1	0.0230 (2)	0.0289 (3)	0.0525 (4)	0.000	0.0066 (2)	0.000
W2	0.0455 (4)	0.0462 (4)	0.0574 (5)	0.000	0.000	0.0195 (3)
Re1	0.0337 (5)	0.0436 (6)	0.0743 (8)	0.000	0.000	−0.0133 (5)
V1	0.0337 (5)	0.0436 (6)	0.0743 (8)	0.000	0.000	−0.0133 (5)
O1C	0.019 (4)	0.017 (5)	0.032 (6)	0.000	0.000	0.000
O1E	0.035 (5)	0.062 (9)	0.108 (12)	0.000	0.000	−0.029 (9)
O2E	0.083 (11)	0.078 (11)	0.082 (10)	0.000	0.000	0.065 (9)
O3E	0.023 (4)	0.046 (6)	0.081 (9)	0.000	0.012 (5)	0.000
O1	0.066 (10)	0.061 (11)	0.016 (6)	0.000	0.000	0.000
O2	0.040 (4)	0.037 (4)	0.063 (5)	0.000 (4)	0.013 (4)	0.006 (4)
O3	0.030 (4)	0.021 (4)	0.076 (8)	0.000	0.000	0.000 (5)
O4	0.019 (2)	0.030 (3)	0.063 (5)	0.000 (3)	0.001 (3)	−0.012 (3)
O5	0.027 (6)	0.047 (9)	0.043 (8)	0.000	0.000	0.000
N1	0.028 (6)	0.076 (12)	0.066 (11)	0.000	−0.006 (6)	0.000
N2	0.029 (5)	0.077 (12)	0.066 (10)	0.000	0.013 (6)	0.000
C1	0.033 (5)	0.053 (7)	0.080 (10)	0.017 (5)	−0.014 (5)	0.006 (7)
C2	0.037 (4)	0.023 (4)	0.069 (8)	0.001 (4)	0.003 (5)	−0.001 (5)
C3	0.029 (5)	0.029 (6)	0.056 (9)	0.000	−0.015 (6)	0.000
O1W	0.034 (7)	0.13 (2)	0.027 (8)	0.000	0.000	0.000
O2W	0.057 (8)	0.109 (14)	0.071 (11)	0.000	0.007 (7)	0.000

### Geometric parameters (Å, º)

**Table:**

O1C—W1	2.2796 (8)	C2—C1	1.339 (15)
O1C—W1^i^	2.2796 (8)	C2—H2	0.9300
O1C—W2^i^	2.286 (9)	C1—N1	1.305 (13)
O1C—W2	2.286 (9)	C1—H1	0.9300
O1C—Re1	2.318 (9)	N1—C1^ii^	1.305 (13)
O1C—V1^i^	2.318 (9)	N1—H1A	0.8600
O1C—Re1^i^	2.318 (9)	O2E—W2	1.707 (12)
O3—W2	1.883 (12)	W1—O4^iii^	1.882 (7)
O3—Re1	1.925 (12)	W1—O2^iii^	1.936 (9)
O1E—Re1	1.674 (14)	W1—Re1^i^	3.2040 (11)
O4—W1	1.882 (7)	W1—Re1	3.2040 (11)
O4—Re1	1.885 (6)	W2—O2^iv^	1.921 (8)
O5—V1^i^	1.958 (9)	W2—Re1	3.2322 (18)
O5—Re1^i^	1.958 (9)	Re1—O4^iv^	1.885 (6)
O5—Re1	1.958 (9)	Re1—W1^i^	3.2040 (11)
O3E—W1	1.718 (10)	N2—H2A	0.8600
O2—W2	1.921 (8)	O1W—H1W1	0.81
O2—W1	1.936 (9)	O2W—H1W2	0.85
O1—W2	1.895 (7)	O2W—H2W2	0.85
O1—W2^i^	1.895 (7)	O3W—H1W3	0.84
C3—N2	1.307 (17)	O4W—H1W4	0.85
C3—C2^ii^	1.414 (12)	O4W—H2W4	0.85
C3—C2	1.414 (12)		
			
W1—O1C—W1^i^	175.3 (6)	O2—W1—Re1	80.8 (2)
W1—O1C—W2^i^	91.6 (2)	O1C—W1—Re1	46.3 (2)
W1^i^—O1C—W2^i^	91.6 (2)	Re1^i^—W1—Re1	62.09 (5)
W1—O1C—W2	91.6 (2)	O2E—W2—O3	101.2 (8)
W1^i^—O1C—W2	91.6 (2)	O2E—W2—O1	105.1 (8)
W2^i^—O1C—W2	90.7 (4)	O3—W2—O1	153.7 (5)
W1—O1C—Re1	88.4 (2)	O2E—W2—O2	103.9 (3)
W1^i^—O1C—Re1	88.4 (2)	O3—W2—O2	87.1 (3)
W2^i^—O1C—Re1	179.9 (4)	O1—W2—O2	86.7 (3)
W2—O1C—Re1	89.19 (5)	O2E—W2—O2^iv^	103.9 (3)
W1—O1C—V1^i^	88.4 (2)	O3—W2—O2^iv^	87.1 (3)
W1^i^—O1C—V1^i^	88.4 (2)	O1—W2—O2^iv^	86.7 (3)
W2^i^—O1C—V1^i^	89.19 (5)	O2—W2—O2^iv^	152.2 (5)
W2—O1C—V1^i^	179.9 (4)	O2E—W2—O1C	179.4 (7)
Re1—O1C—V1^i^	90.9 (4)	O3—W2—O1C	78.1 (4)
W1—O1C—Re1^i^	88.4 (2)	O1—W2—O1C	75.6 (4)
W1^i^—O1C—Re1^i^	88.4 (2)	O2—W2—O1C	76.1 (3)
W2^i^—O1C—Re1^i^	89.19 (5)	O2^iv^—W2—O1C	76.1 (3)
W2—O1C—Re1^i^	179.9 (4)	O2E—W2—Re1	133.6 (7)
Re1—O1C—Re1^i^	90.9 (4)	O3—W2—Re1	32.3 (3)
W2—O3—Re1	116.1 (5)	O1—W2—Re1	121.4 (4)
W1—O4—Re1	116.6 (4)	O2—W2—Re1	80.3 (3)
V1^i^—O5—Re1	115.1 (8)	O2^iv^—W2—Re1	80.3 (3)
Re1^i^—O5—Re1	115.1 (8)	O1C—W2—Re1	45.8 (2)
W2—O2—W1	116.2 (4)	O1E—Re1—O4	102.9 (2)
W2—O1—W2^i^	118.2 (7)	O1E—Re1—O4^iv^	102.9 (2)
N2—C3—C2^ii^	120.6 (6)	O4—Re1—O4^iv^	154.0 (4)
N2—C3—C2	120.6 (6)	O1E—Re1—O3	105.0 (7)
C2^ii^—C3—C2	118.6 (12)	O4—Re1—O3	88.1 (3)
C1—C2—C3	119.0 (11)	O4^iv^—Re1—O3	88.1 (3)
C1—C2—H2	120.5	O1E—Re1—O5	101.5 (7)
C3—C2—H2	120.5	O4—Re1—O5	86.0 (3)
N1—C1—C2	117.1 (12)	O4^iv^—Re1—O5	86.0 (3)
N1—C1—H1	121.4	O3—Re1—O5	153.5 (5)
C2—C1—H1	121.4	O1E—Re1—O1C	178.5 (7)
C1^ii^—N1—C1	128.8 (14)	O4—Re1—O1C	77.0 (2)
C1^ii^—N1—H1A	115.6	O4^iv^—Re1—O1C	77.0 (2)
C1—N1—H1A	115.6	O3—Re1—O1C	76.6 (4)
O3E—W1—O4	104.2 (4)	O5—Re1—O1C	77.0 (4)
O3E—W1—O4^iii^	104.2 (4)	O1E—Re1—W1^i^	134.60 (4)
O4—W1—O4^iii^	89.5 (5)	O4—Re1—W1^i^	122.3 (2)
O3E—W1—O2^iii^	101.6 (4)	O4^iv^—Re1—W1^i^	31.7 (2)
O4—W1—O2^iii^	154.0 (3)	O3—Re1—W1^i^	81.8 (2)
O4^iii^—W1—O2^iii^	87.3 (4)	O5—Re1—W1^i^	79.7 (3)
O3E—W1—O2	101.6 (4)	O1C—Re1—W1^i^	45.33 (2)
O4—W1—O2	87.3 (4)	O1E—Re1—W1	134.60 (4)
O4^iii^—W1—O2	154.0 (3)	O4—Re1—W1	31.7 (2)
O2^iii^—W1—O2	84.4 (5)	O4^iv^—Re1—W1	122.3 (2)
O3E—W1—O1C	176.7 (6)	O3—Re1—W1	81.8 (2)
O4—W1—O1C	78.1 (3)	O5—Re1—W1	79.7 (3)
O4^iii^—W1—O1C	78.1 (3)	O1C—Re1—W1	45.33 (2)
O2^iii^—W1—O1C	76.0 (3)	W1^i^—Re1—W1	90.62 (4)
O2—W1—O1C	76.0 (3)	O1E—Re1—W2	136.5 (6)
O3E—W1—Re1^i^	136.0 (3)	O4—Re1—W2	81.7 (3)
O4—W1—Re1^i^	82.9 (2)	O4^iv^—Re1—W2	81.7 (3)
O4^iii^—W1—Re1^i^	31.75 (19)	O3—Re1—W2	31.5 (3)
O2^iii^—W1—Re1^i^	80.8 (2)	O5—Re1—W2	122.0 (4)
O2—W1—Re1^i^	122.3 (2)	O1C—Re1—W2	45.0 (2)
O1C—W1—Re1^i^	46.3 (2)	W1^i^—Re1—W2	61.16 (3)
O3E—W1—Re1	136.0 (3)	W1—Re1—W2	61.16 (3)
O4—W1—Re1	31.75 (19)	C3—N2—H2A	121
O4^iii^—W1—Re1	82.9 (2)	H1W2—O2W—H2W2	116
O2^iii^—W1—Re1	122.3 (2)	H1W4—O4W—H2W4	116
			
N2—C3—C2—C1	−178.7 (16)	W1^i^—O1C—Re1—W2	−91.7 (2)
C2^ii^—C3—C2—C1	−2 (3)	O3E—W1—Re1—O1E	−5.6 (10)
C3—C2—C1—N1	−1 (2)	O4—W1—Re1—O1E	−3.2 (10)
C2—C1—N1—C1^ii^	6 (3)	O4^iii^—W1—Re1—O1E	97.6 (9)
Re1—O4—W1—O3E	178.3 (5)	O2—W1—Re1—O1E	−102.8 (9)
Re1—O4—W1—O4^iii^	−77.1 (5)	O1C—W1—Re1—O1E	177.8 (9)
Re1—O4—W1—O2^iii^	5.6 (11)	Re1^i^—W1—Re1—O1E	122.9 (9)
Re1—O4—W1—O2	77.0 (5)	O3E—W1—Re1—O4	−2.4 (7)
Re1—O4—W1—O1C	0.7 (4)	O4^iii^—W1—Re1—O4	100.7 (7)
Re1—O4—W1—Re1^i^	−46.0 (4)	O2^iii^—W1—Re1—O4	−177.1 (6)
W2—O2—W1—O3E	179.7 (5)	O2—W1—Re1—O4	−99.7 (6)
W2—O2—W1—O4	−76.3 (5)	O1C—W1—Re1—O4	−179.0 (6)
W2—O2—W1—O4^iii^	6.9 (12)	Re1^i^—W1—Re1—O4	126.1 (5)
W2—O2—W1—O2^iii^	78.9 (5)	O3E—W1—Re1—O4^iv^	179.8 (6)
W2—O2—W1—O1C	2.0 (4)	O4—W1—Re1—O4^iv^	−177.8 (8)
W2—O2—W1—Re1^i^	3.5 (6)	O4^iii^—W1—Re1—O4^iv^	−77.05 (19)
W2—O2—W1—Re1	−45.1 (4)	O2^iii^—W1—Re1—O4^iv^	5.1 (4)
W2^i^—O1C—W1—O4	179.3 (4)	O2—W1—Re1—O4^iv^	82.5 (4)
W2—O1C—W1—O4	88.6 (3)	O1C—W1—Re1—O4^iv^	3.2 (4)
Re1—O1C—W1—O4	−0.5 (3)	Re1^i^—W1—Re1—O4^iv^	−51.7 (3)
V1^i^—O1C—W1—O4	−91.5 (4)	O3E—W1—Re1—O3	97.4 (5)
Re1^i^—O1C—W1—O4	−91.5 (4)	O4—W1—Re1—O3	99.8 (5)
W2^i^—O1C—W1—O4^iii^	−88.6 (3)	O4^iii^—W1—Re1—O3	−159.5 (3)
W2—O1C—W1—O4^iii^	−179.3 (4)	O2^iii^—W1—Re1—O3	−77.3 (4)
Re1—O1C—W1—O4^iii^	91.5 (4)	O2—W1—Re1—O3	0.1 (3)
V1^i^—O1C—W1—O4^iii^	0.5 (3)	O1C—W1—Re1—O3	−79.2 (4)
Re1^i^—O1C—W1—O4^iii^	0.5 (3)	Re1^i^—W1—Re1—O3	−134.1 (2)
W2^i^—O1C—W1—O2^iii^	1.5 (3)	O3E—W1—Re1—O5	−101.6 (6)
W2—O1C—W1—O2^iii^	−89.2 (4)	O4—W1—Re1—O5	−99.2 (6)
Re1—O1C—W1—O2^iii^	−178.3 (4)	O4^iii^—W1—Re1—O5	1.5 (4)
V1^i^—O1C—W1—O2^iii^	90.7 (3)	O2^iii^—W1—Re1—O5	83.7 (4)
Re1^i^—O1C—W1—O2^iii^	90.7 (3)	O2—W1—Re1—O5	161.1 (4)
W2^i^—O1C—W1—O2	89.2 (4)	O1C—W1—Re1—O5	81.8 (4)
W2—O1C—W1—O2	−1.5 (3)	Re1^i^—W1—Re1—O5	26.9 (3)
Re1—O1C—W1—O2	−90.7 (3)	O3E—W1—Re1—O1C	176.6 (6)
V1^i^—O1C—W1—O2	178.3 (4)	O4—W1—Re1—O1C	179.0 (6)
Re1^i^—O1C—W1—O2	178.3 (4)	O4^iii^—W1—Re1—O1C	−80.3 (4)
W2^i^—O1C—W1—Re1^i^	−89.14 (5)	O2^iii^—W1—Re1—O1C	1.9 (4)
W2—O1C—W1—Re1^i^	−179.9 (4)	O2—W1—Re1—O1C	79.3 (4)
Re1—O1C—W1—Re1^i^	91.0 (4)	Re1^i^—W1—Re1—O1C	−54.9 (3)
V1^i^—O1C—W1—Re1^i^	0.0	O3E—W1—Re1—W1^i^	179.0 (5)
W2^i^—O1C—W1—Re1	179.9 (4)	O4—W1—Re1—W1^i^	−178.6 (5)
W2—O1C—W1—Re1	89.14 (5)	O4^iii^—W1—Re1—W1^i^	−77.9 (2)
V1^i^—O1C—W1—Re1	−91.0 (4)	O2^iii^—W1—Re1—W1^i^	4.3 (3)
Re1^i^—O1C—W1—Re1	−91.0 (4)	O2—W1—Re1—W1^i^	81.7 (3)
Re1—O3—W2—O2	−76.4 (2)	O1C—W1—Re1—W1^i^	2.4 (3)
Re1—O3—W2—O2^iv^	76.4 (2)	Re1^i^—W1—Re1—W1^i^	−52.53 (4)
W2^i^—O1—W2—O2	76.5 (3)	O3E—W1—Re1—W2	122.8 (5)
W2^i^—O1—W2—O2^iv^	−76.5 (3)	O4—W1—Re1—W2	125.2 (5)
W1—O2—W2—O2E	177.3 (8)	O4^iii^—W1—Re1—W2	−134.1 (2)
W1—O2—W2—O3	76.5 (6)	O2^iii^—W1—Re1—W2	−51.9 (3)
W1—O2—W2—O1	−78.0 (6)	O2—W1—Re1—W2	25.5 (3)
W1—O2—W2—O2^iv^	−1.5 (16)	O1C—W1—Re1—W2	−53.8 (3)
W1—O2—W2—O1C	−2.0 (4)	Re1^i^—W1—Re1—W2	−108.71 (2)
W1—O2—W2—Re1	44.6 (4)	O2—W2—Re1—O1E	100.0 (3)
W1—O1C—W2—O2	1.5 (3)	O2^iv^—W2—Re1—O1E	−100.0 (3)
W1^i^—O1C—W2—O2	178.2 (4)	O2E—W2—Re1—O4	−100.0 (2)
Re1—O1C—W2—O2	89.9 (3)	O3—W2—Re1—O4	−100.0 (2)
W1—O1C—W2—O2^iv^	−178.2 (4)	O1—W2—Re1—O4	80.0 (2)
W1^i^—O1C—W2—O2^iv^	−1.5 (3)	O2—W2—Re1—O4	−0.1 (3)
Re1—O1C—W2—O2^iv^	−89.9 (3)	O2^iv^—W2—Re1—O4	160.0 (3)
W1—O4—Re1—O1E	177.7 (7)	O1C—W2—Re1—O4	80.0 (2)
W1—O4—Re1—O4^iv^	4.3 (15)	O2E—W2—Re1—O4^iv^	100.0 (2)
W1—O4—Re1—O3	−77.4 (5)	O3—W2—Re1—O4^iv^	100.0 (2)
W1—O4—Re1—O5	76.8 (6)	O1—W2—Re1—O4^iv^	−80.0 (2)
W1—O4—Re1—O1C	−0.7 (4)	O2—W2—Re1—O4^iv^	−160.0 (3)
W1—O4—Re1—W1^i^	1.6 (6)	O2^iv^—W2—Re1—O4^iv^	0.1 (3)
W1—O4—Re1—W2	−46.3 (4)	O1C—W2—Re1—O4^iv^	−80.0 (2)
W2—O3—Re1—O4	77.1 (2)	O2E—W2—Re1—O3	0.000 (2)
W2—O3—Re1—O4^iv^	−77.1 (2)	O1—W2—Re1—O3	180.000 (2)
W2—O3—Re1—W1^i^	−45.92 (4)	O2—W2—Re1—O3	100.0 (3)
W2—O3—Re1—W1	45.92 (4)	O2^iv^—W2—Re1—O3	−100.0 (3)
V1^i^—O5—Re1—O4	−77.6 (2)	O1C—W2—Re1—O3	180.000 (2)
Re1^i^—O5—Re1—O4	−77.6 (2)	O2E—W2—Re1—O5	180.000 (1)
V1^i^—O5—Re1—O4^iv^	77.6 (2)	O3—W2—Re1—O5	180.000 (1)
Re1^i^—O5—Re1—O4^iv^	77.6 (2)	O1—W2—Re1—O5	0.000 (1)
V1^i^—O5—Re1—W1^i^	46.26 (5)	O2—W2—Re1—O5	−80.0 (3)
Re1^i^—O5—Re1—W1^i^	46.26 (5)	O2^iv^—W2—Re1—O5	80.0 (3)
V1^i^—O5—Re1—W1	−46.26 (5)	O1C—W2—Re1—O5	0.000 (1)
Re1^i^—O5—Re1—W1	−46.26 (5)	O2E—W2—Re1—O1C	180.0
W1—O1C—Re1—O4	0.5 (3)	O3—W2—Re1—O1C	180.0
W1^i^—O1C—Re1—O4	177.2 (4)	O1—W2—Re1—O1C	0.0
V1^i^—O1C—Re1—O4	88.9 (3)	O2—W2—Re1—O1C	−80.0 (3)
Re1^i^—O1C—Re1—O4	88.9 (3)	O2^iv^—W2—Re1—O1C	80.0 (3)
W1—O1C—Re1—O4^iv^	−177.2 (4)	O2E—W2—Re1—W1^i^	125.752 (18)
W1^i^—O1C—Re1—O4^iv^	−0.5 (3)	O3—W2—Re1—W1^i^	125.752 (18)
V1^i^—O1C—Re1—O4^iv^	−88.9 (3)	O1—W2—Re1—W1^i^	−54.248 (18)
Re1^i^—O1C—Re1—O4^iv^	−88.9 (3)	O2—W2—Re1—W1^i^	−134.3 (3)
W1—O1C—Re1—O5	−88.3 (2)	O2^iv^—W2—Re1—W1^i^	25.8 (3)
W1^i^—O1C—Re1—O5	88.3 (2)	O1C—W2—Re1—W1^i^	−54.248 (18)
W1—O1C—Re1—W1^i^	−176.7 (4)	O2E—W2—Re1—W1	−125.752 (18)
V1^i^—O1C—Re1—W1^i^	−88.3 (2)	O3—W2—Re1—W1	−125.752 (18)
Re1^i^—O1C—Re1—W1^i^	−88.3 (2)	O1—W2—Re1—W1	54.248 (18)
W1^i^—O1C—Re1—W1	176.7 (4)	O2—W2—Re1—W1	−25.8 (3)
V1^i^—O1C—Re1—W1	88.3 (2)	O2^iv^—W2—Re1—W1	134.3 (3)
Re1^i^—O1C—Re1—W1	88.3 (2)	O1C—W2—Re1—W1	54.248 (18)
W1—O1C—Re1—W2	91.7 (2)		

Symmetry codes: (i) −*x*+1/2, −*y*, *z*; (ii) *x*, −*y*+1, *z*; (iii) *x*, −*y*, *z*; (iv) −*x*+1/2, *y*, *z*.

### Hydrogen-bond geometry (Å, º)

**Table:**

*D*—H···*A*	*D*—H	H···*A*	*D*···*A*	*D*—H···*A*
N1—H1*A*···O1*W*	0.86	2.40	3.078 (14)	136
N1—H1*A*···O1*E*	0.86	2.58	3.184 (14)	129
N2—H2*A*···O3^v^	0.86	2.39	3.181 (12)	152
O1*W*—H1*W*1···O1*E*	0.81	2.50	3.188 (18)	144
O2*W*—H1*W*2···O3*E*^vi^	0.85	2.10	2.836 (18)	144
O4*W*—H1*W*4···O2*W*	0.85	1.87	2.40 (7)	119
O4*W*—H2*W*4···O4*W*^vii^	0.85	2.31	2.65 (15)	106

Symmetry codes: (v) *x*+1/2, *y*, −*z*+1; (vi) −*x*+1, −*y*, −*z*+1; (vii) −*x*+1, −*y*, −*z*+2.
